# Seven myths of randomisation in clinical trials

**DOI:** 10.1186/1745-6215-12-S1-A95

**Published:** 2011-12-13

**Authors:** Stephen Senn

**Affiliations:** 1 CRP Santé, Strassen, L-1445, Luxembourg

## 

Randomisation in clinical trials continues to be controversial and to be attacked on ethical, practical and philosophical grounds. Whatever the practical, moral or philosophical limitations of randomisation may be, many of the attacks reveal that the critics do not understand why randomisation is carried out and what its purpose is. In some cases one may even claim that the defenders of randomisation are equally confused.

Seven common myths are examined in this paper, namely, that 1. patients are treated simultaneously in clinical trials 2. balance of prognostic factors in necessary for valid inference 3. observed covariates may be ignored because one has randomized 4. blinding can be carried out effectively without randomisation 5. randomisation is inefficient 6. randomisation precludes balancing covariates and 7. large trials are more balanced than small ones.

Many of these myths are related to the problem of distinguishing between conditional and unconditional inference and the relevance of this distinction is explained with a simple game of chance involving two fair dice, and played in three variants, and a statistician who has to correctly call the odds of obtaining a total score of ten. Others simply arise, because many who have written on clinical trials have not designed them.

In fact, what some critics have overlooked is that when it comes to allocating treatments to patients, ‘the devil is in the detail’. A useful discipline that can be recommended to any would-be critic of randomisation in clinical trials is to attempt to write a detailed protocol, capable of being followed by another. This ‘thought experiment’ is useful in revealing potential problems with any scheme.

Finally a technical problem to do with the estimation of nuisance parameters in analysis of covariance is covered.

It is concluded that the debate on the role of randomised clinical trials in evidence based medicine would be improved if those debating it paid careful attention to what randomisation can and cannot do to strengthen the validity of inferences regarding the effects of treatment.

